# NEDD4-1 deficiency impairs satellite cell function during skeletal muscle regeneration

**DOI:** 10.1186/s40659-023-00432-7

**Published:** 2023-05-05

**Authors:** Felipe Cabezas, Claudio Cabello-Verrugio, Natalia González, Jeremy Salas, Manuel J. Ramírez, Eduardo de la Vega, Hugo C. Olguín

**Affiliations:** 1 Laboratory of Tissue Repair and Adult Stem Cells, Molecular and Cell Biology Department, Faculty of Biological Sciences, Pontificia Universidad Católica de Chile, Santiago, Chile; 2 Departamento de Ciencias Biológicas y Químicas, Facultad de Medicina y Ciencia, Universidad San Sebastián, Lota 2465, 7510157 Santiago, Chile; 3 Laboratory of Muscle Pathology, Fragility and Aging, Faculty of Life Sciences, Universidad Andres Bello, Santiago, Chile; 4 Millennium Institute on Immunology and Immunotherapy, Faculty of Life Sciences, Universidad Andres Bello, Santiago, Chile

**Keywords:** Skeletal muscle regeneration, Muscle differentiation, Satellite cells, NEDD4-1, Muscle stem cells

## Abstract

**Background:**

Satellite cells are tissue-specific stem cells primarily responsible for the regenerative capacity of skeletal muscle. Satellite cell function and maintenance are regulated by extrinsic and intrinsic mechanisms, including the ubiquitin–proteasome system, which is key for maintaining protein homeostasis. In this context, it has been shown that ubiquitin-ligase NEDD4-1 targets the transcription factor PAX7 for proteasome-dependent degradation, promoting muscle differentiation in vitro. Nonetheless, whether NEDD4-1 is required for satellite cell function in regenerating muscle remains to be determined.

**Results:**

Using conditional gene ablation, we show that NEDD4-1 loss, specifically in the satellite cell population, impairs muscle regeneration resulting in a significant reduction of whole-muscle size. At the cellular level, NEDD4-1-null muscle progenitors exhibit a significant decrease in the ability to proliferate and differentiate, contributing to the formation of myofibers with reduced diameter.

**Conclusions:**

These results indicate that NEDD4-1 expression is critical for proper muscle regeneration in vivo and suggest that it may control satellite cell function at multiple levels.

**Supplementary Information:**

The online version contains supplementary material available at 10.1186/s40659-023-00432-7.

## Background

The ability to regenerate or repair damaged tissues is crucial to maintain the homeostasis and survival of different organisms. In vertebrates, the skeletal muscle is a dynamic tissue with a high capacity for adaptation upon use and regeneration after injury [7, 11, 50]. This regenerative response is sustained mainly by a set of tissue-specific stem cells, called satellite cells (SCs), that reside between the basal lamina and the muscle fiber plasma membrane or sarcolemma [28]. Under homeostatic conditions, SCs exist in a non-proliferative state, expressing the paired box transcription factor PAX7, essential for SCs specification and maintenance [19, 36, 41, 47, 49]. Upon extrinsic stimuli, such as muscle injury, SCs become activated and proliferate extensively, inducing the expression of MYF5 and MYOD, members of the Muscle Regulatory Factor (MRFs) family of transcription factors [9, 48, 53]. Later, the induction of the MRF Myogenin marks an irreversible step toward terminal differentiation and cell fusion, repairing damaged fibers or forming new myofibers [1, 13]. During this process, SCs self-renew, maintaining the pool of quiescent SCs after muscle regeneration is complete [14, 24, 29]. Among different molecular pathways regulating SC function, controlling protein homeostasis via the ubiquitin–proteasome system (UPS) has gained recent attention [35]. The UPS mediates highly specific intracellular protein degradation and is crucial for several eukaryotes processes [15]. Accordingly, a recent study showed that SC-specific proteasome dysfunction impaired SC function in vitro and in vivo [22].

Protein ubiquitination involves the covalent attachment of the small protein ubiquitin to lysine residues of substrate proteins, which then acts as a molecular tag [40, 51]. Three major sets of proteins are required for ubiquitination: (1) E1 ubiquitin-activating enzyme, (2) E2 ubiquitin-conjugating enzyme, and (3) E3 ubiquitin ligase [39]. Activated ubiquitin is transferred to the target protein by the ubiquitin E3 ligase (which can additionally catalyze the formation of polyubiquitin chains) and thus are critical for UPS specificity. In this context, Bustos and cols. [5] showed that E3 ubiquitin ligase NEDD4-1 (neural precursor cell expressed developmentally down-regulated protein 4–1) ubiquitinates Pax7 inducing its proteasome-dependent degradation in the myogenic cell line C2C12, promoting muscle differentiation. NEDD4-1 is a ubiquitously expressed member of the HECT (Homologous to the E6-AP Carboxyl Terminus) family of E3 ubiquitin ligases [43] which regulates membrane, cytoplasmic and nuclear proteins affecting a variety of cellular processes [3, 18, 52]. Although few studies have investigated NEDD4-1 function in skeletal muscle, *nedd4-1*^*−/−*^ mice exhibit underdeveloped neuromuscular junctions [26]. Conditional *nedd4-1* deletion in adult myofibers prevents short-term loss of muscle mass upon denervation [33] but not upon other atrogenic stimuli. Finally, it has been shown that NEDD4-1 could regulate muscle size via NOTCH1 inactivation in rat skeletal muscles [23].

Although these results indicate that NEDD4-1 participates in diverse skeletal muscle signaling pathways, it remains to be determined if NEDD4-1 is specifically required for regulating SC regenerative potential in vivo.

Here, we present evidence indicating that SC-specific *nedd4-1* conditional deletion in adult mice severely impairs muscle regeneration resulting in the formation of myofibers with significantly reduced diameter, the increase in extracellular matrix deposition, and the decrease of whole muscle size. *Nedd4-1*-null muscle progenitors exhibited diminished proliferation and differentiation capacity, providing a cellular mechanism for the reduced regenerative potential.

## Results

### NEDD4-1 expression is dynamically regulated in myogenic cells during regeneration

**Fig. 1:**
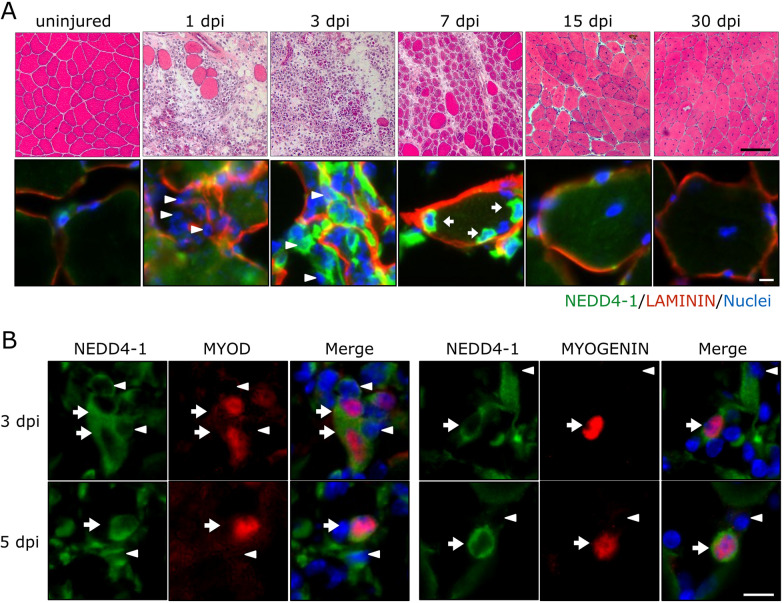
NEDD4-1 expression during skeletal muscle regeneration. **A** Nedd4-1 expression during skeletal muscle regeneration at different days post injury (dpi) with BaCl_2_ (1.2% w/v). Upper panels show H&E staining of TA cryo-sections. Degenerating myofibers together with abundant mononuclear cells were observed at 1–3 dpi, while muscle cytoarchitecture was progressively recovered by 30 dpi. Scale bar = 100 μm. Lower panels show representative immunofluorescence of TA cryo-sections, marked with anti-NEDD4-1 (green), LAMININ (red), and DAPI for nuclear staining (blue). Regenerating myofibers exhibit centrally located nuclei. NEDD4-1 expression peaked at 3–7 dpi and decreased by 30 dpi at levels comparable to uninjured muscle. Arrowheads indicate interstitial cells; arrows indicate Nedd4(+) cells closely associated with myofiber’s basal lamina. Scale bar = 20 μm. **B** Double immunofluorescence for NEDD4-1 and MYOD (left panel) and NEDD4-1 and MYOGENIN (right panel) at 3 and 5 dpi. Arrowheads indicate non-myogenic NEDD4-1(+) cells; arrows indicate myogenic cells co-expressing MYOD or MYOGENIN and NEDD4-1. DAPI was used for nuclear staining (blue). Scale bar = 20 μm

To evaluate the role of NEDD4-1 in SC function in vivo, we first analyzed NEDD4-1 expression pattern in activated SCs upon induced muscle injury and regeneration. For this, tibialis anterior (TA) muscles from adult mice were injured by barium chloride (BaCl_2_) intramuscular injection, and NEDD4-1 expression was evaluated by indirect immunofluorescence (IF) at different days post-injury (dpi). As described previously [5], low levels of NEDD4-1 were detected in cells located underneath the myofiber basal lamina in uninjured muscles (Fig. 1A, uninjured). Noteworthy, low levels of NEDD4-1 were consistently detected also in a sub-sarcolemmal pattern, which is in line with previous studies [23]. Concomitantly with disorganization of muscle architecture and increased cellular infiltration, NEDD4-1 expression was detected in the interstitial cell population at 1 and 3 dpi (Fig. 1A). At 7 dpi, cells expressing high levels of NEDD4-1 were detected in association with the basal lamina of regenerating fibers (Fig. 1A). By 15 and 30 dpi, NEDD4-1 levels gradually decreased to those detected in uninjured muscle (Fig. 1A). Since we observed the peak of NEDD4-1 expression between 3 and 7 dpi, we determined the myogenic nature of the NEDD4-1(+) cells. At 3 and 5 dpi, a sub-population of NEDD4-1(+) cells also expressed myogenic markers, such as MyoD and Myogenin (Fig. 1B). These observations indicate that NEDD4-1 expression is dynamic during muscle regeneration and is expressed in myogenic and non-myogenic cells during active tissue remodeling, gradually becoming restricted to the myogenic lineage during myofiber formation/growth.

**Fig. 2:**
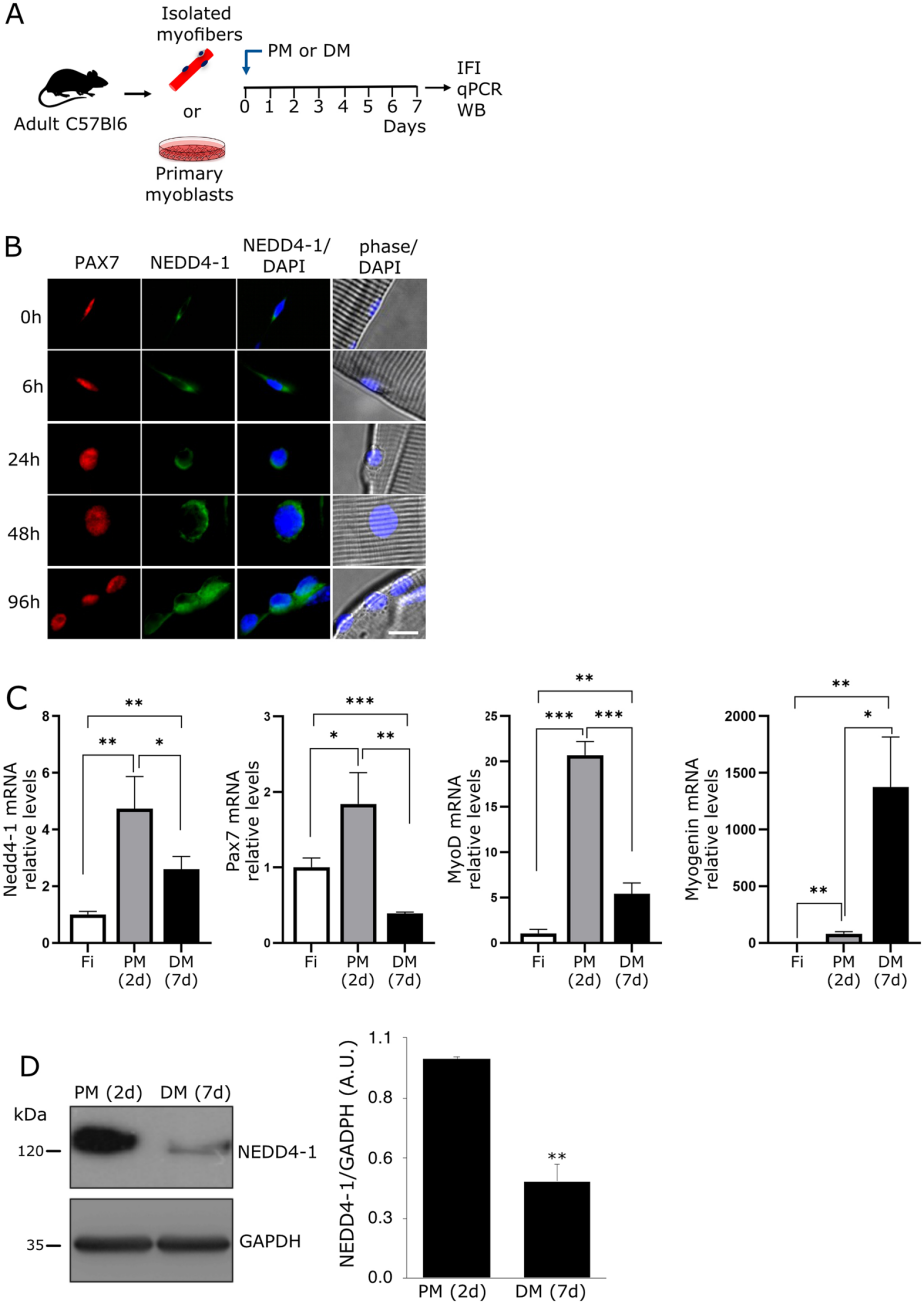
Nedd4-1 kinetics expression in activated and differentiated SCs. **A** Skeletal muscle fibers or SCs were isolated from wild-type c57/bl6 adult mice and maintained in proliferation (PM) or differentiation (DM) culture conditions. At the indicated time points, Nedd4-1 expression was evaluated by IF, qPCR, or Western blot (WB). **B** Isolated skeletal muscle fibers were maintained in proliferation conditions, and NEDD4-1 expression in associated SCs was analyzed by IF (green) at the indicated time points. PAX7 expression was used as SCs marker (red). Nuclei were marked by DAPI staining (blue). Scale bar = 10 μm. **C** Isolated SCs were maintained in PM for 2 days (2d) or DM for 7 days (7d), and Nedd4-1 mRNA relative levels were measured by qPCR. Pax7, MyoD, and Myogenin mRNA levels were measured to monitor myogenic progression. Freshly isolated (Fi) SCs were used to determine mRNA basal levels. 18S RNA was used as housekeeping for normalization. Results were expressed as the average RQ ± SD; n = 3. *P-value < 0.05; **P-value < 0.01, ***, P-value < 0.001. **D** Western blot shows the expression of NEDD4-1 in SCs cultures maintained in PM and DM for 2 and 7 days, respectively. For quantitative analysis, GAPDH expression was used for normalization in arbitrary units (A.U); n = 3, **P value < 0.01

NEDD4-1 expression in myogenic progenitors was further evaluated ex-vivo during SC activation on single myofiber cultures (Fig. 2A). As determined by IF, NEDD4-1 was expressed in Pax7(+) cells associated to freshly isolated myofibers (Fig. 2B, 0 h). NEDD4-1 levels gradually increased at later time points post isolation (Fig. 2B, 6–96 h). qPCR analyses on SCs primary cultures corroborated these observations: compared to freshly isolated SCs, there was a ≥ threefold increase in Nedd4-1 mRNA levels after 48 h in proliferating culture conditions (Fig. 2C). MyoD mRNA levels also increased by 48 h (Fig. 2C), consistent with SCs activation and proliferation. When cells were maintained in differentiation culture conditions for 7 days, Nedd4-1 mRNA levels decreased significantly (~ 50%), although they remained elevated compared to freshly isolated cells. As expected, Pax7 and MyoD expression decreased after 7 days in differentiation culture conditions, contrasting with the significant induction of Myogenin mRNA (Fig. 2C). This decrease in Nedd4-1 expression upon differentiation was also observed at the protein level, as determined by Western blot (Fig. 2D). Since NEDD4-1 appeared to be expressed in non-myogenic cells in vivo, we corroborated the myogenic enrichment of our primary cultures, analyzing the expression of the fibroblast marker Tcf4 [27] by qPCR. Myogenic and non-myogenic subpopulations were differentially enriched using the pre-plating method described elsewhere [42]. Under such conditions, Tcf4 mRNA was highly expressed in the fibroblast-enriched cell fraction, while the myogenic fraction expressed almost undetectable levels of Tcf4 (Additional file 1: Fig. S1A). Enrichment of each cell population was further corroborated by phase contrast microscopy at 2 and 7 days in culture (Additional file 1: Fig. S1B). Together, these results indicate that Nedd4-1 expression is dynamically regulated during SC activation and myogenic progression.

### Conditional *nedd4-1* ablation in satellite cells impairs muscle regeneration

**Fig. 3:**
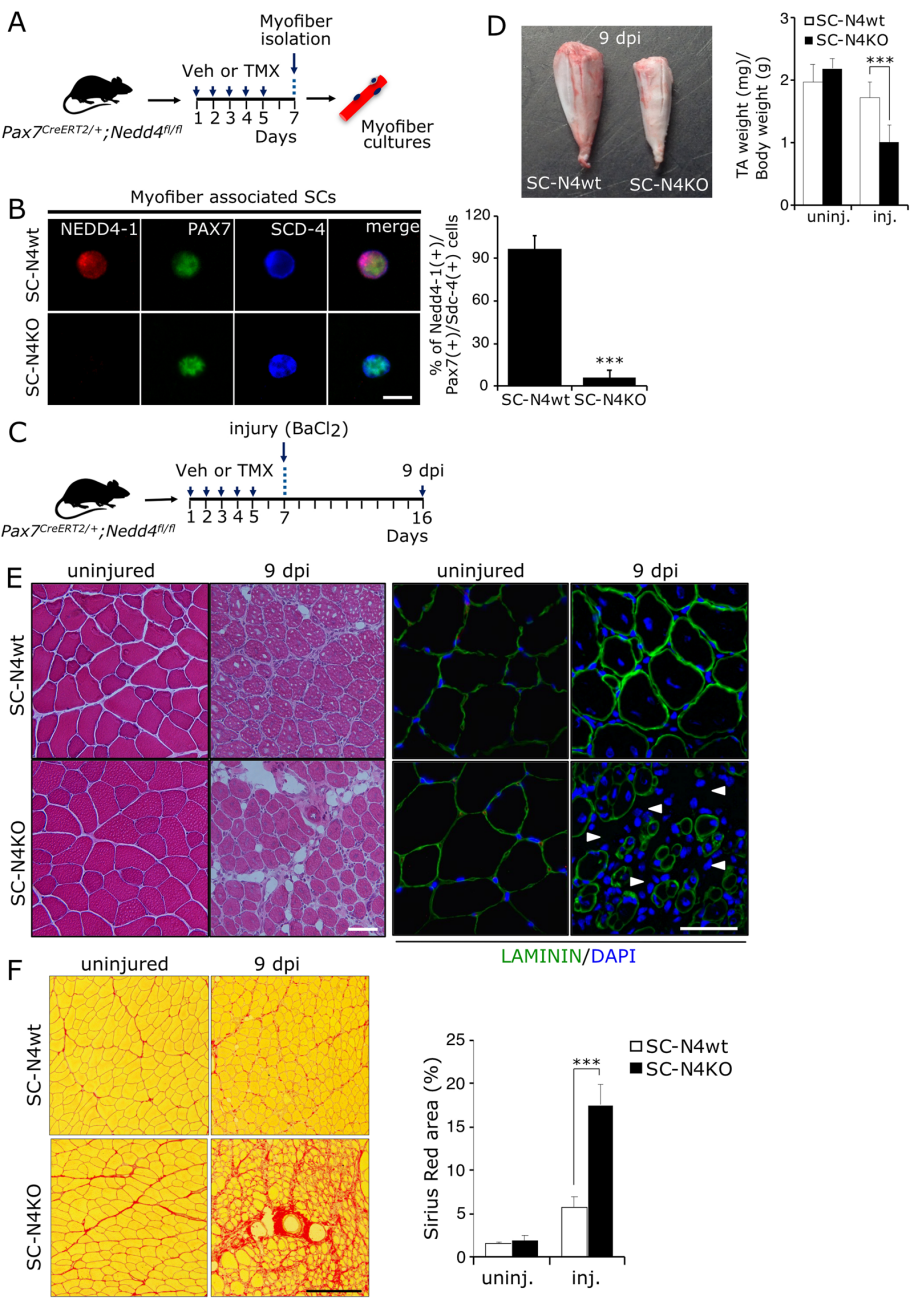
Effect of Nedd4-1 deletion in SCs during early muscle regeneration. **A** Pax7^*CreERT2/*+^*; Nedd4-1*^*f/f*^ mice were injected with tamoxifen (TMX) or vehicle (Veh) for five days (see “Materials and methods” section). 48 h after the final dose, myofibers were isolated for further analysis. **B** Myofibers were isolated from mice treated as in **A** and maintained in proliferation culture conditions for 24 h. NEDD4-1 expression was analyzed by IF. PAX7 and SYNDECAN-4 (Sdc-4) were used as independent SC markers. Quantification shows average percentage of NEDD4-1(+)/PAX7(+)/SYNDECAN-4(+) cells upon treatment with vehicle (SC-N4wt) or TMX (SC-N4KO); n = 3, ***P-value < 0.001. Scale bar: 10 μm. **C** *Pax7*^*CreERT2/*+^*; Nedd4-1*^*f/f*^ mice were injected with tamoxifen (TMX) or vehicle (Veh) for five days. 48 h after the final dose, TA muscles were injured by BaCl_2_ intramuscular injection (see “Materials and methods” section). Injured and contra-lateral uninjured TAs were isolated at 9 dpi for further analysis. **D** Representative image of frozen injured TAs obtained from SC-N4wt and SC-N4KO animals, respectively. Quantification shows normalized TA weight of contra-lateral (uninj.) and injured (inj.) muscles isolated from SC-N4wt (white bars) and SC-N4KO (black bars) animals; n = 3, ***P-value < 0.001. **E** H&E staining (left panels) of injured and contra-lateral (uninjured) TA cryosections from SC-N4wt and SC-N4KO animals. Dotted lines highlight differences in muscle size/shape. IF staining (right panels) for LAMININ (green) also shows differences in tissue architecture. Arrowheads indicate areas devoid of myofibers/enriched in infiltrating cells. Scale bars: 50 μm. **F** Sirius red staining of injured (9 dpi) and contra-lateral (uninjured) TA cryosections. Quantification of total Sirius red-stained area shows increased collagen deposition in injured SC-N4KO muscles; n = 3, ***P < 0.001. Scale bar: 200 μm

As shown above, NEDD4-1 expression is dynamic in the regenerating muscle tissue in myogenic and non-myogenic cell populations. This precludes directly determining NEDD4-1 function in the SCs. Therefore, we developed the *Pax7*^*CreERT2/*+^; *Nedd4-1*^*fl/fl*^ transgenic mouse model (see “Materials and methods” section) to conditionally eliminate *Nedd4-1* expression in SCs from adult animals. To test the efficiency of induced NEDD4-1 loss, animals were subject to 5 daily injections of tamoxifen (TMX) or vehicle (see “Materials and methods” section). Single myofibers were isolated from TA muscles and maintained in proliferation culture conditions for 24 h before fixation and analysis of SYNDECAN-4 (general SC marker), PAX7, and NEDD4-1 expression by IF (Fig. 3A). While ~ 95% of myofiber-associated SCs from control animals (SC-N4wt) stained positive for all three markers, NEDD4-1 expression was detected in ~ 5% of SYNDECAN-4(+)/PAX7(+) cells, obtained from animals treated with TMX (SC-N4KO; Fig. 3B). Next, we evaluated the effect of SC-NEDD4-1 loss on skeletal muscle regeneration upon acute muscle injury. For this, injury by BaCl_2_ injection was performed two days after TMX treatment. Both injured and contra-lateral uninjured TA muscles were collected initially at 9 dpi (Fig. 3C), after the peak of NEDD4-1 expression (see Fig. 1A). Compared to SC-N4wt (*Pax7*^*CreERT2/*+^; *Nedd4-1*^+*/*+*)*^, SC-N4KO (*Pax7*^*CreERT2/*+^; *Nedd4-1*^*fl/fl*^) injured TAs exhibited > 40% reduction in weight upon isolation (Fig. 3D). Histological analysis of tissue architecture showed no significant differences between uninjured SC-N4KO and SC-N4wt contra-lateral muscles (Fig. 3E, left panels). Regenerating myofibers (characterized by centrally located myonuclei) were observed in SC-N4wt injured TAs by H&E staining (Fig. 3E, left panels). However, SC-N4KO injured TAs exhibited increased tissue disorganization, including areas populated by myofibers with small diameters compared to SC-N4wt and areas where myofibers were absent (Fig. 3E, left panels). This was further confirmed by IF, where LAMININ expression was used to delineate muscle fiber basal lamina, revealing areas of regenerating myofibers with reduced diameter and increased interstitial cellular infiltrate (Fig. 3E, right panels). Given the differences in tissue organization, we evaluated if the increased areas lacking muscle fibers in SC-N4KO injured tissue correlated with changes in matricellular protein deposition. Indeed, Sirius Red staining revealed a ~ 3.5-fold increase in collagen-rich total area compared to SC-N4wt (Fig. 3F).

**Fig. 4:**
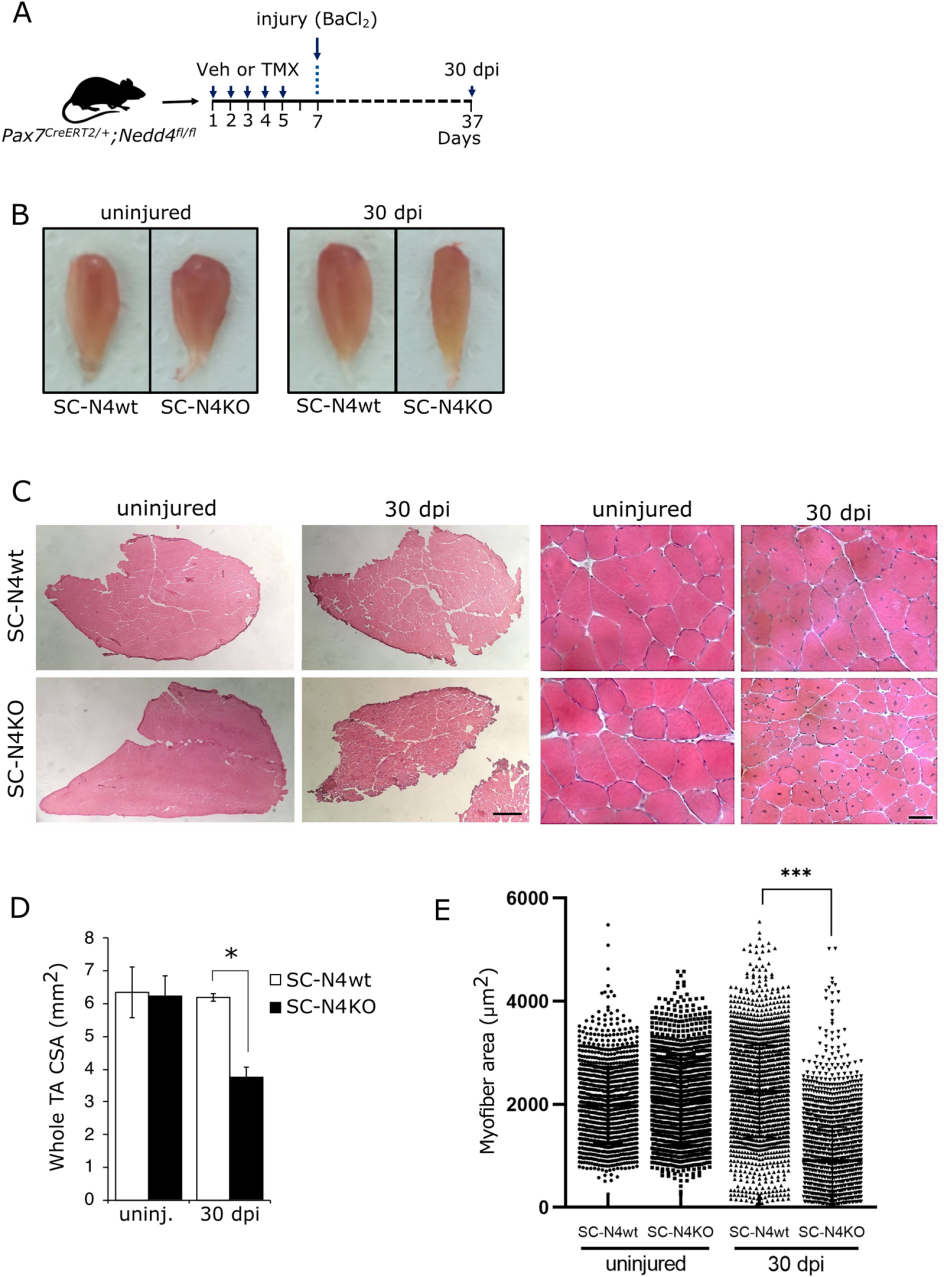
Effect of Nedd4-1 deletion in SCs during late muscle regeneration. **A** *Pax7*^*CreERT2/*+^*; Nedd4-1*^*f/f*^ mice were injected with tamoxifen (TMX) or vehicle (Veh) for five days. 48 h after the final dose, TA muscles were injured by BaCl_2_ intramuscular injection (see materials and methods). Injured and contra-lateral uninjured TAs were isolated at 30 dpi for further analysis. **B** Comparison of freshly isolated contra-lateral (uninjured) and injured (30 dpi) TA muscle treated as in **A** with vehicle (SC-N4wt) or tamoxifen (SC-N4KO) prior to muscle injury. **C** H&E staining of cryosections from TA muscles obtained as in **A** at × 4 (left panels) and × 40 (right panels) magnification. Scale bars = 500 μm and 40 μm, respectively. **D** Differences in average whole TA cross-sectional area (CSA) between contra-lateral (uninj.) and injured (30 dpi) obtained from SC-N4wt and SC-N4KO animals; n = 3, *P < 0.05. **E** Quantification shows individual fiber CSA for regenerating (centrally located nuclei) myofibers. Uninjured contralateral myofiber CSA are shown as internal control. mean values are indicated for each condition. n = 3, ***P < 0.001

Next, we evaluated the effect of SC-specific NEDD4-1 loss at later stages of muscle regeneration. For this, TA muscles were injured as described and collected at 30 dpi (Fig. 4A). As observed previously, no significant differences in whole TA size were detected between uninjured SC-N4wt and SC-N4KO upon isolation; however, injured SC-N4KO exhibited a noticeable mass reduction when compared to injured SC-N4wt (Fig. 4B). H&E staining on tissue sections corroborated a ~ 45% reduction on average whole TA cross-sectional area (CSA) (Fig. 4C, D). Closer inspection revealed that overall tissue architecture was restored in both SC-N4wt and SC-N4KO TAs; however, fiber size was significantly reduced upon NEDD4-1 loss in SCs, as indicated by myofiber CSA distribution (Fig. 4E). Myofiber CSA distribution showed no significant differences comparing uninjured TA muscles from both genotypes. Together, these results suggest that in the absence of NEDD4-1, SC regenerative function is severely compromised, leading to decreased myofiber and whole-muscle size.

### NEDD4-1 loss in satellite cells decreases the expansion and differentiation of myogenic progenitors

**Fig. 5:**
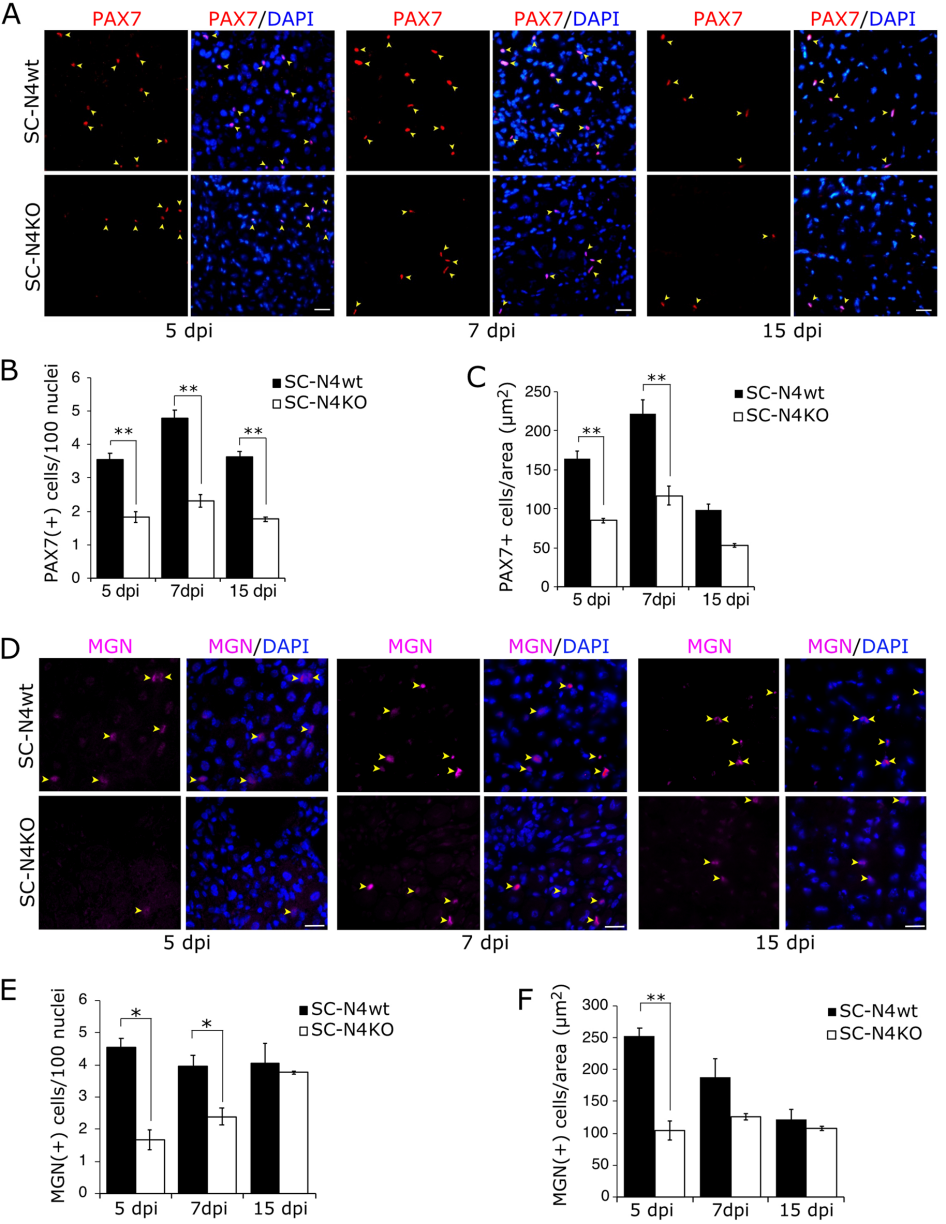
Nedd4-1 ablation results in decreased expansion and differentiation of myogenic progenitors in vivo. **A** SC-N4wt and SC-N4KO TA muscles were obtained at 5, 7, and 15 days post-injury (dpi), cryosectioned, and analyzed by IF for PAX7 expression (red; arrowheads). Nuclei were stained with DAPI (blue). Scale bar = 30 μm. **B** Number of myogenic progenitors (PAX7 +) per 100 nuclei from cryosections obtained as in **A**; n = 3; **P-value < 0.01. **C** Number of myogenic progenitors (PAX7+) per section area (mm^2^) from cryosections obtained as in **A**; n = 3; **P-value < 0.01. **D** SC-N4wt and SC-N4KO TA muscles obtained as in **A** were cryosectioned and analyzed by IF for MYOGENIN (MGN) expression (magenta, arrowheads). Nuclei were stained with DAPI (blue). Scale bar = 20 μm. **E** Number of myogenic progenitors (MGN+) per 100 nuclei from cryosections obtained as in **D**; n = 3, *P-value < 0.05. **F** Number of myogenic progenitors (MGN+) per section area (mm^2^) from cryosections obtained as in **D**; n = 3, **P-value < 0.01

We hypothesized that impaired regeneration and reduced muscle mass observed in SC-N4KO upon injury could arise at least by two mechanisms: (a) defective expansion of progenitors with myogenic potential or (b) defective differentiation of myogenic progenitors. We tested these concepts first by analyzing the expression of PAX7 (as a marker for activated/proliferating progenitors) and MYOGENIN (as a marker for differentiating myogenic cells) at different time points during regeneration upon BaCl_2_-induced injury. Using IF, we observed a significant reduction in the number of PAX7(+) cells at 5, 7, and 15 dpi in SC-N4KO injured TAs (Fig. 5A). Considering the previously observed differences in whole-muscle size between SC-N4wt and SC-N4KO regenerating TAs, the number of PAX7(+) cells was normalized to the number of nuclei (Fig. 5B) or section area (Fig. 5C), resulting in a similar ~ twofold reduction. Using a similar approach, the number of MYOGENIN(+) cells appear significantly reduced at early time points in SC-N4KO compared to SC-N4wt (Fig. 5D). Quantification of the number of MYOGENIN(+) cells, normalized by the number of nuclei (Fig. 5E) or section area (Fig. 5F) showed a ~ threefold reduction in SC-N4KO compared to SC-N4wt. This difference gradually declined at 7 dpi and was not observed by 15 dpi, which is consistent with the cellular dynamics reported previously, where phases of muscle progenitor proliferation, differentiation, cell fusion, and myofiber growth are finely orchestrated during regeneration [2].

**Fig. 6:**
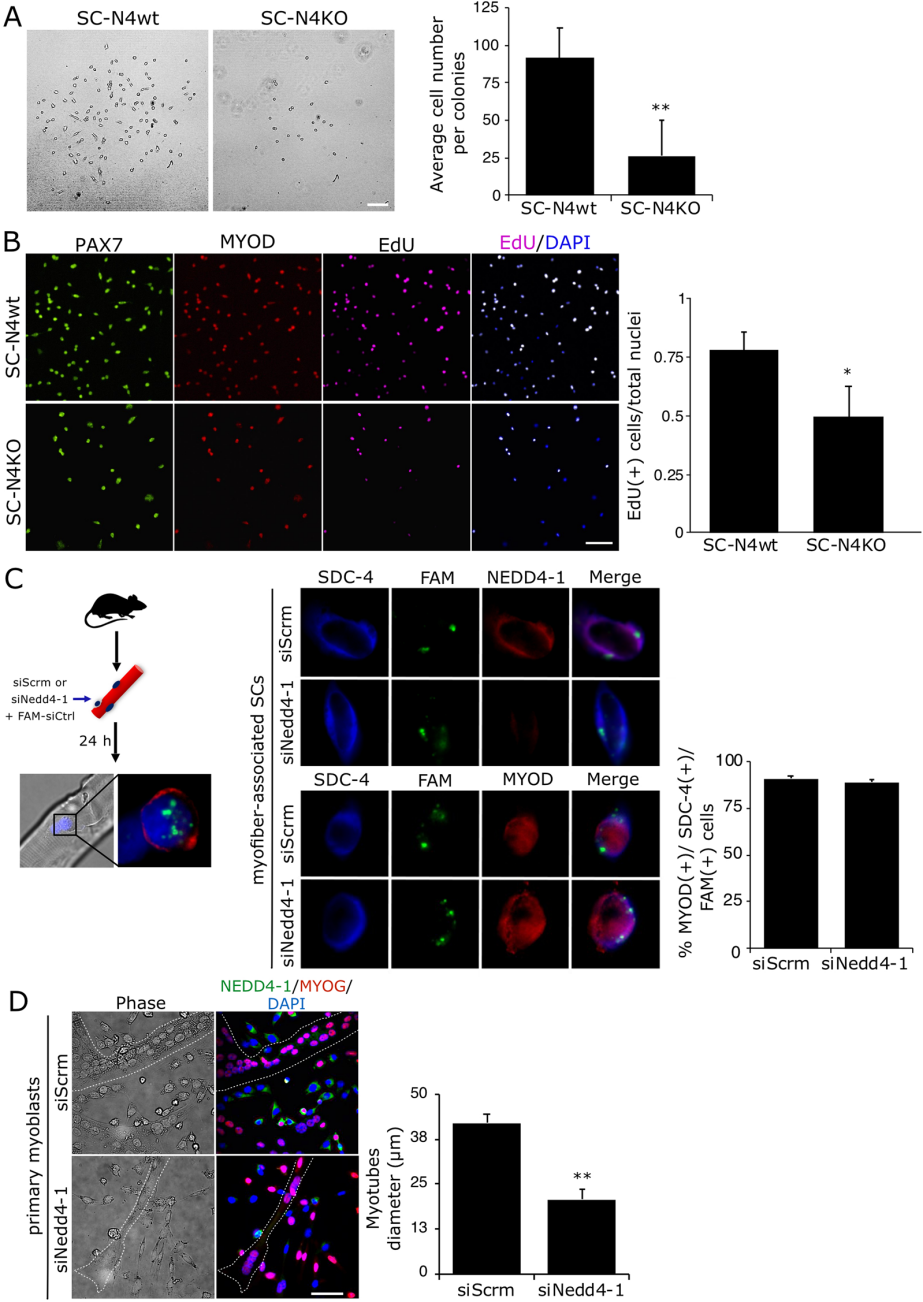
Nedd4-1 ablation results in decreased expansion and differentiation of myogenic progenitors in vitro. **A** Representative images of primary myoblasts colonies by phase contrast microscopy from SC-N4wt and SC-N4KO mice, maintained in proliferation conditions for 48 h. Quantification shows the average number of cells per colony (n = 3, **P < 0.01). Scale bar = 100 μm. **B** Primary myoblasts isolated from SC-N4wt and SC-N4KO mice and maintained in proliferation conditions were labeled with EdU for 6 h prior to fixation. EdU (magenta) incorporation and PAX7 (green) or MYOD (red) expression were analyzed by IF. Quantification shows the average number of myogenic EdU(+) cells, normalized by the total number of cells, in each condition; n = 3, *P < 0.05. Scale bar = 100 μm. **C** Single myofiber-associated SCs were transfected upon isolation with control (siScramble) or Nedd4-1 targeting siRNA (siNedd4-1) for 24 h prior to fixation and IF. Transfection was monitored by co-transfection with a FAM-labeled non-targeting siRNA (FAM; green). SYNDECAN-4 (Sdc-4; blue), NEDD4-1 (red, upper panel), and MYOD (red, lower panel) were detected by IF. Quantification shows the percentage of MYOD(+)/SDC-4(+)/FAM(+) cells in each condition. n = 3. **D** Primary myoblasts obtained and treated as in (I) were maintained in differentiating culture conditions for 72 h. NEDD4-1 and MYOGENIN (Myog) expression were analyzed by IF (green and red, respectively). Nuclei were marked with DAPI staining. Dotted lines trace the periphery of multinucleated cells identified by phase contrast. Quantification shows the average myotube diameter in each condition. n = 3, **P < 0.01

Together, our previous results suggest that the loss of NEDD4-1 expression compromises the expansion of myogenic progenitors. To determine if this effect was cell autonomous, primary myoblasts cultures from SC-N4wt and SC-N4KO muscles were isolated and maintained in proliferation medium for 24–48 h. Under these conditions, SC-N4KO myoblasts consistently formed colonies with significantly fewer cells than SC-N4wt myoblasts (Fig. 6A). This effect was exacerbated after sequential passaging, where we observed fewer adherent SC-N4KO myoblasts compared to SC-N4wt (Additional file 2: Fig. S2A, B). Noteworthy, the adherent SC-N4KO population were homogeneously PAX7(+) cells (Additional file 2: Fig. S2C).

Next, we evaluated the proliferative status of SC-N4wt and SC-N4KO primary myoblasts by EdU incorporation assay (see Materials and methods). As determined by IF, EdU was detected mainly in PAX7(+)/MYOD(+) cells in both SC-N4wt and SC-N4KO (Fig. 6B), confirming their muscle progenitor status. Quantification showed a ~ 25% reduction in the number of SC-N4KO cells labeled with EdU compared to SC-N4wt cells, indicating lower proliferative capacity in *Nedd4-1*-null myoblasts.

Since NEDD4-1 loss had a marked impact on cell numbers, we initially tested the effect on differentiation by siRNA-mediated *Nedd4-1* knockdown. For this, myofiber-associated SCs were transfected with *Nedd4-1* specific siRNA (siNedd4-1) or a non-targeting siRNA (siScrm) upon isolation for 24 h prior to fixation and IF (Fig. 6C, left panel). Using the SC marker SYNDECAN-4 SC, we evaluated the expression of MYOD and NEDD4-1 (Fig. 6C, middle panel). Quantification showed no effect of acute NEDD4-1 knockdown on MYOD expression levels, in line with the results described above, indicating no effect on SC activation. Next, siNedd4-1 and siScrm transfected SCs were seeded on gelatin-coated dishes and maintained in differentiating culture conditions for 72–96 h prior to fixation. As determined by phase contrast microscopy and IF, siNedd4-1 transfected cells induced the expression of the early differentiation marker Myogenin but formed a reduced number of myotubes with decreased average diameters (Fig. 6D). Together, these results suggest that NEDD4-loss also impairs terminal differentiation in muscle progenitors.

**Fig. 7:**
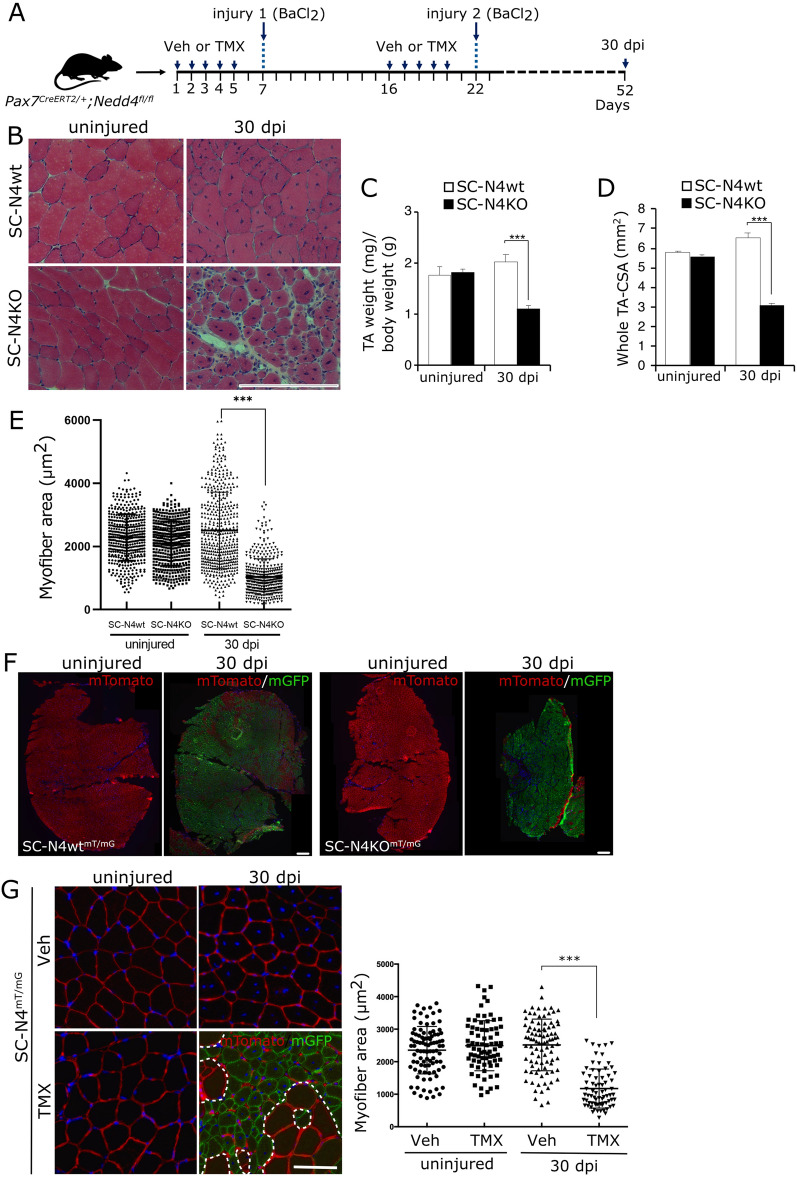
Effect of Nedd4-1 ablation in SC upon repeated injury and regeneration. **A** Schematic of TMX and BaCl_2_ treatment to induce consecutive injury in TA muscles. Samples were collected 30 days after the second injury (30 dpi; 52 days) for further analysis. **B** H&E staining of contra-lateral (uninjured) and injured (30 dpi) TAs from SC-N4wt and SC-N4KO mice. Scale bar = 200 μm. **C** Quantification shows the average TA weights ± SEM (normalized by total body weight) from SC-N4wt (white) and SC-N4KO (black bars) at 30 dpi. **D** Quantification shows the average whole TA CSA ± SEM from SC-N4wt (white) and SC-N4KO (black bars) at 30 dpi. For **C** and **D**, n = 3, ***P < 0.001. **E** Quantification shows individual fiber CSA for regenerating (centrally located nuclei) myofibers. Uninjured contralateral myofiber CSA are shown as internal control. mean values are indicated for each condition. n = 3, ***P < 0.001. **F** TA cryosections from SC-N4wt^*mT/mG*^ and SC-N4KO^*mT/mG*^ Cre-reporter mice after 30 days of BaCl_2_-induced injury (30 dpi). mGFP(+) myofibers show contribution of recombined SCs to muscle regeneration. Representative of 3 independent experiments. Scale bar = 200 μm. **G** Differences in regenerating diameter from SC-N4^*mT/mG*^ Cre-reporter mice treated with vehicle (veh; centrally located nuclei non-recombined GFP−) or tamoxifen (TMX; centrally located nuclei/GFP+). Quantification shows individual fiber CSA, and mean values are indicated for each condition. n = 3, ***P < 0.001. Scale bar = 100 μm

As an indirect measure of the capacity for self-renewal, we evaluated the ability of *Nedd4*-null SCs to respond upon consecutive injury events. For this, two successive cycles of injury and regeneration were performed in SC-N4wt and SC-N4KO. Briefly, TMX or vehicle injection followed by BaCl_2_-induced injury was performed as described previously; at 10 dpi, the second round of vehicle or TMX treatment was initiated, followed by a second intramuscular BaCl_2_ injection. 30 days after the second injury, muscles were collected, fixed, and cryosectioned (Fig. 7A). H&E staining showed a noticeable difference between SC-N4wt and SC-N4KO in myofiber size and overall tissue architecture (Fig. 7B). Quantification of TA weight indicated a ~ 50% reduction in SC-N4KO compared to SC-N4wt (Fig. 7C). At the same time, whole muscle CSA was reduced by ~ twofold in SC-N4KO (Fig. 7D), compared to ~ 1.5 reduction after single injury (see Fig. 4D).

As shown previously, individual fiber CSA distribution indicates a significant decrease in SC-N4KO myofiber size (Fig. 7E). Noteworthy, high heterogeneity in myofiber size was corroborated by IF (Additional file 3: Fig. S3, right panel), which also showed a high presence of interstitial cells in SC-N4KO regenerating muscles when compared to SCN4wt, accompanied by increased deposition of collagen-rich extracellular matrix, as determined by Sirius red staining (Additional file 3: Fig. S3, left panel).

NEDD4 loss significantly affected the proliferation and differentiation of muscle precursors, particularly in vitro, which raised the question about the origin of the regenerating myofibers detected after injury in SC-N4KO muscles. Particularly considering that after TMX-induced recombination, ~ 5% of cells retain NEDD4-1 expression (see Fig. 3B) and could be responsible for partial regeneration. To address this, we generated a Cre-reporter model using the *ROSA*^*mT/mG*^ line (SC-N4^mT/mG^ henceforth, see “Materials and methods” section), where CreERT2-mediated recombination activates the expression of membrane-bound GFP. This allows effective tracing of recombined SCs and their descendants. SC-N4wt^mT/mG^ and SC-N4KO^mT/mG^ TA muscles were injured by BaCl_2_ injection and collected at 30 dpi to determine mGFP expression by IF. As expected, SC-N4wt^mT/mG^ exhibit a large proportion of GFP(+) myofibers (Fig. 7F), indicating the robust regenerative potential of mGFP-expressing SCs. As shown previously, SC-N4KO^mT/mG^ exhibited a marked reduction in whole muscle size, but most regenerating fibers expressed mGFP (Fig. 7F), tracing their origin to *Nedd4-1*-null SCs. Consistent with our previous results, quantifying the average cross-sectional area of individual regenerating myofibers showed a significant reduction (~ 1.5-fold) in SC-N4KO^mT/mG^, compared to regenerating myofibers of injured SC-N4KO^mT/mG^ where recombination was not previously induced (Fig. 7G; TMX vs. veh). Similar experiments indicate that reduction in whole muscle size and heterogeneous myofiber diameter were still observed in N4KO TAs at 60 dpi, suggesting long-term defects in muscle regeneration (Additional file 4: Fig. S4). Together, these results indicate that *Nedd4*-null SCs can activate upon injury in vivo; however, their regenerative capacity is impaired, resulting in the formation of smaller myofibers and reduced muscle size.

## Discussion

Control of protein homeostasis via the ubiquitin–proteasome system (UPS) appears critical for SC function [22, 35]. In this context, we previously showed that E3 ubiquitin ligase NEDD4-1 can regulate the activation of the differentiation program in muscle progenitors in vitro [5]. Here we evaluated the requirement of NEDD4-1 for the regulation of SC regenerative potential in vivo*,* via inducible SC-specific *Nedd4* loss.

First, we showed that NEDD4-1 is dynamically expressed during muscle regeneration. Between 0 and 3 dpi, NEDD4-1 expression increased and was observed in both myogenic and non-myogenic infiltrating cells. Based on the extensive descriptions regarding the temporality and different cell types involved in muscle regeneration, non-myogenic cells expressing NEDD4-1 are likely inflammatory cells, mainly macrophages [2], where *Nedd4-1* expression is crucial for their function [25, 37]. Between 5 and 7 dpi, NEDD4-1 became gradually restricted to the myogenic lineage (correlating with a decreasing non-muscle cellular infiltrate), specifically in cells expressing the early differentiation marker MYOGENIN. Intriguingly, at ~ 7 dpi, high NEDD4-1 expression remains associated with sub-laminar nuclei in regenerating fibers but not centrally located myonuclei, potentially marking fusing muscle progenitors. By 15 dpi, very low levels of NEDD4-1 are detected in myofibers which becomes almost undetectable by 30 dpi. NEDD4-1 protein levels correlate with mRNA levels, suggesting that *Nedd4-1* expression is controlled at the transcriptional level in activated SCs and committed muscle progenitors. The apparent down-regulation of *Nedd4-1* expression in regenerated muscle agrees with a study by Nagpal [33].

The same study shows that *Nedd4-1* expression is induced in muscle upon denervation. Moreover, muscle-specific deletion of *Nedd4-1* protects the denervated muscle from atrophy. Intriguingly, Nedd4-1 expression is not induced in response to other atrophy-inducing perturbations, such as immobilization. These observations suggest that *Nedd4-1* plays critical and distinct roles in the myogenic lineage, depending on the differentiation status and environmental context.

Based on our studies, we hypothesized that NEDD4-1 loss in the SC population could have detrimental effects on the regeneration response. Indeed, compared to control mice, regenerating SC-N4KO muscles exhibited a significant reduction in whole muscle weight and cross-sectional area, which correlates with a marked decrease in myofiber diameter. Our in-vivo and ex-vivo observations suggest two cellular processes underlying this phenotype. First, *Nedd4-1*-null cells showed diminished proliferation capacity. Defective expansion of muscle progenitors results in decreased cells available for differentiation and myofiber formation [4]. NEDD4-1 loss could potentially impact several pathways controlling proliferation in muscle progenitors. Our previous work showed that NEDD4-1 targets PAX7 for proteasome-dependent degradation allowing induction of the differentiation program [5]. NEDD4-1 loss could lead to increased PAX7 stability and accumulation in activated SCs.

Interestingly, higher levels of PAX7 decreased proliferation and prevented differentiation in myogenic cell lines [34]. Indeed, Western blot analyses from total muscle extracts at 3, 5 and 9 dpi indicate elevated PAX levels, despite the reduction in the total number of PAX7-expressing cells in regenerating SC-N4KO muscles (data not shown). On the other hand, Nedd4-1 has been shown to regulate Notch signaling in different cellular contexts [23, 44]. Since activation of the NOTCH pathway has been involved in the regulation of quiescence and proliferation of muscle progenitors, it is plausible that NEDD4-1 loss could affect their expansion by a NOTCH-dependent mechanism. Further studies are required to define the molecular mechanism(s) involved in NEDD4-1-dependent control of muscle progenitor proliferation.

The second process potentially underlying the observed regeneration phenotype is the effect of NEDD4-1 in terminal differentiation and myofiber formation. We previously showed that acute Nedd4-1 knockdown in primary myoblasts impairs their ability to form multi-nucleated myotubes [5]. Here we complemented those observations and showed that Nedd4-1 knockdown in myofiber-associated SCs did not affect MYOD (i.e., SC activation). Nonetheless, Nedd4-1 downregulation results in the formation of fewer myotubes with reduced diameters. Interestingly, Myogenin induction was not significantly affected by siNedd4-1 treatment in vitro, suggesting that Nedd4-1 could regulate differentiation downstream of MRF expression.

The decrease in whole muscle size could be primarily explained by the significant reduction in myofiber cross-sectional area, which could result from diminished muscle progenitor proliferation and differentiation, as discussed above. However, since Nedd4-1 is expressed in myofibers (albeit at lower levels), we cannot rule out the possibility that Nedd4-1 deficiency may impair muscle growth. Since the control of muscle mass is mainly achieved by opposite regulation of the rate of protein synthesis v/s. protein degradation [45], the requirement of controlled protein degradation to increase myofiber mass appears as an attractive area that requires further investigation. Moreover, it has been suggested that Nedd4-1 could control animal growth via IGF signaling [6, 12]. In this context, Nedd4-1 function in skeletal muscle has been studied only in atrophy models (with contradictory results), whereas its role during muscle growth/hypertrophy remains unexplored.

Our regeneration studies show no significant effect on contra-lateral uninjured muscles, indicating no major impact on short-term muscle maintenance. Considering recent long-term studies demonstrating the differential incorporation of SCs into resting muscles [21] and their controversial requirement for muscle hypertrophy [30, 31], it would be interesting to study the effect of NEDD4-1 loss in SCs under similar conditions. Moreover, human correlational studies suggest the involvement of Nedd4-1 in sarcopenia during aging [10].

## Conclusions

Ubiquitin ligase NEDD4-1 is expressed in activated satellite cells in vivo. Genetic ablation of Nedd4-1 expression in satellite cells impairs muscle regeneration in response to acute injury. *Nedd4-1*-null muscle progenitors exhibit a context-dependent proliferation and differentiation capacity reduction, suggesting that Nedd4-1 could regulate satellite cell function at multiple levels.

## Methods

### Mice

*Nedd4-1* SC-specific conditional knockout mice (*Pax7*^*CreERT2/+*^; *Nedd4-1*^*fl/fl*^) were obtained after breeding* Nedd4-1*^*fl/fl*^, gently provided by Dr. Hiroshi Kawabe [20], with the *B6.Cg-Pax7*^*CreERT2*^ mouse line [32], obtained from Jackson Laboratories (USA). For Nedd4-1 expression kinetic experiments, adult mice (2–4 months old) of strain c57bl/6 were used. For the SC tracing in vivo, *Pax7Cre*^*ERT2/+*^; *Nedd4-1*^*fl/fl*^ mice were bred with B6.129*(Cg)-Gt(ROSA)26Sor*^*tm4(ACTB-tdTomato,-EGFP)Luo*^*/J* reporter mice, obtained from Jackson Laboratories (USA).

### Tamoxifen injection and barium chloride (BaCl_2_) injury

To induce recombination, 2–4 months old *Pax7Cre*^*ERT2/+*^; *Nedd4-1*^*fl/fl*^ mice, were treated with daily doses of 0.1 mg/g for 5 consecutive days with tamoxifen (Sigma-Aldrich, USA) dissolved in 90%v/v sesame oil (Sigma-Aldrich, USA) and 10% v/v ethanol at 25 mg/ml (Merck, Germany), delivered by intraperitoneal injection. To induce acute damage, tibialis anterior (TA) muscles were injured with 60 µl of 1.2% BaCl_2_ diluted in sterile saline (NaCl 0.9%) and delivered via intramuscular injection as described previously [8]. As a control, the contra-lateral TA was injected with an equivalent volume of sterile saline. At indicated days after injury, mice were euthanized, and TAs were removed, frozen in liquid nitrogen-chilled isopentane, and stored at − 80 °C. All animal procedures were performed according to National Commission for Science and Technology (CONICYT) guidelines and approved by the School of Biological Sciences and the Pontificia Universidad Católica de Chile Bioethics and Biosecurity Committee.

### Histological analysis

Muscle histology/architecture and collagen depositions were analyzed by Hematoxylin–Eosin (H&E) and Sirius red (Sigma-Aldrich, USA) staining, respectively, on TA transverse sections. For H&E staining, cryosections (6 µm thickness) were sequentially rehydrated in Phosphate Buffered Saline-1X (PBS-1X) for 5 min, fixed in formalin (10% v/v) for 10 min, and stained using H&E (Merk, Germany) according to manufacturer's instructions. Then, samples were dehydrated and mounted with Entellan mounting medium (Millipore Sigma, USA). For Sirius red staining, slides containing the cryosections were fixed in 100% ethanol at 4 °C for 30 min, incubated with a solution of saturated picric acid at 50 °C for 1 h, washed with distilled water, and incubated with 0,1% Sirius red in saturated picric acid protected from light for 5 min. Then, sections were washed with 2% acetic acid, dehydrated, and mounted with Entellan mounting medium. Sections were analyzed using bright field microscopy on Nikon Eclipse E600. Quantification of myofiber cross-sectional area (CSA) and total Sirius Red positive area were determined using the ImageJ 1.48v software [46].

### Primary myoblasts and isolated muscle fiber cultures

Satellite cell-derived myoblasts were obtained as described [34]. Briefly, hindlimb muscles were dissected from adult mice and digested with 800 U/ml collagenase type I (Worthington, USA) diluted in F12-C medium, supplemented with 1% Penicillin/Streptomycin (P/S), filtered through a 0.22 μm filter, for 45 min at 37 °C with agitation. Next, the digestion mix was diluted with 20 ml of F12-C medium supplemented with 15% horse serum (HS) and 1% P/S, and SC-containing fractions were obtained after sequential filtering of the digested muscles through 70 and 40 μm cell strainer (Thermo Fisher Scientific, USA), followed by centrifugation at 1000x*g* for 10 min. The cell pellet was resuspended in proliferation medium (PM: F12-C supplemented with 15% HS, 1% P/S, and 500 pM FGF-2) and pre-plated onto a plastic culture dish for 1 h to remove remaining fibroblasts. Finally, the SC-enriched supernatants were plated onto collagen coated-dishes and cultured in PM at 37 °C, 6% O_2_ and 5% CO_2_. Differentiation was induced by switching PM to differentiation medium (DM: F12-C supplemented only with 15% HS and 1% P/S). Isolated myofibers were obtained from TAs of adult mice, as previously described [9]. Briefly, TAs were dissected and treated with 400 U/ml collagenase type I in F12-C medium supplemented with 1% P/S at 37 °C for 45 min. Then, myofibers were mechanically separated using a glass Pasteur pipette in PM, and isolated myofibers were fixed or cultured in PM at 37 °C, 6% O_2_, and 5% CO_2_.

### Tissue sections immunostaining

Cryosections obtained from TAs were fixed in paraformaldehyde 4% v/v (PFA 4%) diluted in PBS-1X for 20 min and permeabilized with 0.2%Triton X-100/PBS-1X for 10 min at room temperature. Then, cryosections were incubated with blocking buffer (BB: 5% BSA/PBS-1X) for 1 h and incubated with primary antibody at 4 °C overnight. Samples were then washed with BB and incubated with secondary antibody, and Hoechst 33342 (Thermo Fisher Scientific, USA) diluted in BB at room temperature for 2 h. Samples were then washed with PBS-1X and mounted in Fluoromount-G (Thermo Fisher Scientific, USA). Antigen retrieval was performed before Pax7 immunostaining [17]. Primary antibodies were used at the following dilutions: rabbit polyclonal anti-Nedd4-1 (Abcam, UK) at 1:1000; rat monoclonal anti-Laminin (Sigma-Aldrich, USA) at 1:2000; rabbit polyclonal anti-Myogenin M-225 (Santa Cruz Biotechnology, USA), at 1:200; goat polyclonal anti-Myogenin (Santa Cruz Biotechnology, USA) at 1:500; and rat monoclonal anti-MyoD (EMD Millipore, Germany), at 1:800; chicken anti-Syndecan-4 [8], at 1:500,and mouse monoclonal anti-Pax7 (Developmental Studies Hybridoma Bank, USA), at 1:1. Secondary antibodies used were the following: donkey anti-rat Alexa Fluor 488; donkey anti-rabbit Alexa Fluor 555; donkey anti-goat Alexa Fluor 555; donkey anti-rabbit Alexa Fluor 488; donkey anti-mouse Alexa Fluor 488; donkey anti-mouse Alexa Fluor 594. All these secondary antibodies were used at 1:500 and purchased from Life Technologies (Thermo Fisher Scientific, USA). Secondary antibody, donkey anti-chicken-AMCA (Jackson IR, USA), was used at 1:500 dilution.

### siRNA transfection and in vitro tamoxifen treatment

For knockdown, isolated SCs were plated onto collagen coated-dishes as described, and immediately transfected with Nedd4-1 siRNAs-pool (QIAGEN, USA) or siControl RISC-free siRNAs (Dharmacon, USA) and FAM-labeled Negative control 1 siRNA (5'-carboxyfluorescein label, Thermo Fisher) for 24 or 72 h, using the TransIT-X2® system (Mirus, USA) according to manufacturer’s instructions. For in vitro tamoxifen (TMX) treatments, freshly isolated SCs were incubated with 10 µM TMX in PM, for 12–72 h, followed by fixation and indirect immunofluorescence (IF).

### RNA isolation, reverse transcription and qPCR

Total RNA was extracted using RNA-Solv isolation system (Omega Bio-Tek, USA), following manufacturer's instructions. Total RNA fractions were incubated with DNAse I at room temperature for 15 min, and RNA concentration was quantified using NanoDrop equipment. cDNA was synthesized by reverse transcription, using 0.2 µg of RNA in 20 µl reaction mixture containing random primers, RiboLock RNase inhibitor, and RevertAidTM H Minus M-MuLV (Fermentas, USA). qPCR reactions were performed using SYBR Green master mix (Fermentas, USA) according to the manufacturer's instructions and 7500 Real-Time PCR System (Applied Biosystems, USA). The sequences of primers used for qPCR were as follows: mouse MyoD FW 5’-CACGACTGCTTTCTTCACCA-3’, RV 5’-CGGAACCCCAACAGTACAAT-3’; mouse Pax7 FW 5’-CACCCCTTTCAAAGACCAAA-3’, RV 5’-TGCTTGAAGTTCCTGCTCCT-3’; mouse 18 s 5’-GAGCGAAAGCATTTGCCAAG-3’, RV 5’-GGCATCGTTTATGGTCGGAA-3’; mouse TCF4 FW 5’-GTGCCCGGATGTGAATGGAT-3’, RV 5’-ATCCTCCTCCCCAACACCAT-3; mouse Nedd4-1 FW 5’-GGAGGACGAGGTATGGGAGT-3’, RV 5’-CCAGGTACGGATCAGCAGTG-3’ and mouse Myogenin FW 5’-ATTGTCTGTCAGGCTGGGTG-3’, RV 5’-TAAATTCCCTCGCTGGGCTG-3’.

### Western blotting

Muscle tissues and cultured cells were lysed in RIPA lysis buffer (50 mM Tris–HCl pH 7.4, 150 mM NaCl, plus protease inhibitors) using a 3 ml tissue grinder (Wheaton, USA) and incubated at 4 °C for 30 min, followed by centrifugation at 15,200 rpm for 10 min. Then, the supernatant was collected, and the total proteins were quantified using BCA protein assay kit (Thermo Fisher Scientific, USA). Electrophoresis and western blot were performed as described previously [16]. Briefly, 25 µg of total protein was loaded into 10% sodium dodecyl sulfate polyacrylamide gel electrophoresis (SDS-PAGE) and transferred to polyvinylidene difluoride (PVDF) membranes (Thermo Fisher Scientific, USA). Membranes were blocked with 3% BSA in TBS-T (20 mM Tris, pH 7.4; 100 mM NaCl; 0,5% tween-20) and incubated with the following primary antibodies and dilutions: rabbit polyclonal anti-NEDD4-1 (Abcam), at 1:10,000; mouse monoclonal anti-Myosin Heavy Chain (MHC), at 1:5; mouse monoclonal anti-PAX7, at 1:5; mouse monoclonal (F5D) anti-MYOGENIN, at 1:5 (Developmental Studies Hybridoma Bank, USA) and mouse monoclonal anti-GAPDH (EMD Millipore, Germany), at 1:10,000. Anti-mouse IgG and anti-rabbit IgG Horseradish Peroxidase (HRP) conjugated secondary antibodies (Cell Signaling, USA) were used at 1:5000, and HRP activity was detected using SuperSignal West Pico Chemiluminescent Substrate or SuperSignal West Dura Extended Duration Substrate (Thermo-Fisher Scientific, USA). Western blots signals (n = 3 each condition) were quantified by densitometric analysis, using the ImageJ 1.48v software.

### EdU incorporation assay

Isolated fibers were cultured in PM for 4 days, followed by 48 h culture onto collagen-coated dishes containing collagen-coated glass coverslips. SCs adhered to the collagen substrate were incubated with 10 µM EdU for 6 h and immediately fixed and permeabilized. EdU detection was performed using the Click-iT™ EdU Alexa Fluor™ 647 Imaging Kit (Thermo-Fisher Scientific, USA), following the manufacturer’s instructions. Slides were analyzed using an Andor EMCCD camera attached to a Nikon Eclipse Ti inverted microscope with a Lambda DG4 wavelength-switch xenon light source (Sutter Instruments, USA) controlled by NIS software (Nikon, Japan). Images were analyzed with ImageJ 1.48v processing software.

### Statistical analysis

Western blots densitometry analyses were performed using ImageJ 1.48v software. qPCR analysis was performed using a relative quantification mathematical model, as previously described [38]. All data were expressed as the mean ± standard error of the mean (s.e.m.) from at least three independent experiments. Comparisons > 2 groups were performed using analysis of variance (ANOVA) followed by the Bonferroni correction, and for 2 groups, data were analyzed by Mann Whitney test. All the statistical analyses were performed using GraphPad Prism 5 software (www.graphpad.com).

## Supplementary Information

**Additional file 1: Figure S1.** A) To determine the purity of SCs cultures, the fibroblast marker Tcf4 mRNA relative levels were measured by qPCR from SCs primary cultures maintained in differentiation conditions for 7 days and compared with fibroblasts cultures maintained for the same period. Results were expressed as the average RQ ± SD of three experimental replicates. B) Phase contrast images representative from primary cultures in. Scale bar = 20 μm. Representative of 3–4 independent experiments.

**Additional file 2: Figure S2.** A) Pax7CreERT2/+; Nedd4-1f/f mice were injected with tamoxifenor vehiclefor 5 days. 48 h after the final dose, myofibers were isolated and maintained in proliferating culture conditions for 24 h. Subsequently, culture supernatant was transferred to a second plateand maintained in proliferating culture conditions for 24 h. B) Representative phase contrast microscopy from primary myoblasts obtained as in. n = 3. Scale bar = 10 μm. C) SCs were isolated from Pax7CreERT2/+; Nedd4-1f/f mice and treated with vehicleor TMX. After 72 h, cells were fixed, and NEDD4-1 expression was analyzed by IF. PAX7 expression was used as muscle progenitor marker. Nuclei were stained with DAPI. Arrows indicate NEDD4-1muscle precursors, and asterisk shows non-myogenic cell expressing NEDD4-1 in TMX-treated cultures. Scale bar = 50 μm.

**Additional file 3: Figure S3.** Sirius red staining and IF for lamininof contra-lateraland injuredTAs from SC-N4wt and SC-N4KO mice obtained as in Fig. 6. Scale bars = 200 μm, 50 μm.

**Additional file 4: Figure S4.** A) Normalized TA weightdifferences from SC-N4wtmT/mG and SC-N4KOmT/.mG at 60 dpi. *** P < 0.001. B) TA cryosections from SC-N4wtmT/mG and SC-N4KOmT/mG at 60 dpi. mGFPmyofibers show contribution of recombined SCs to muscle regeneration. Representative of n=3 for SC-WT and n=4 for SC-N4KO. Scale bar= 10 μm.

## Data Availability

All data generated or analyzed during this study are included in this published article (and its additional files).
