# Supplementary figures and images for: NEDD4-1 deficiency impairs satellite cell function during skeletal muscle regeneration
Source: Biol Res. 2023 May 5;56:21. doi: 10.1186/s40659-023-00432-7 (PMC10161651; doi:10.1186/s40659-023-00432-7)

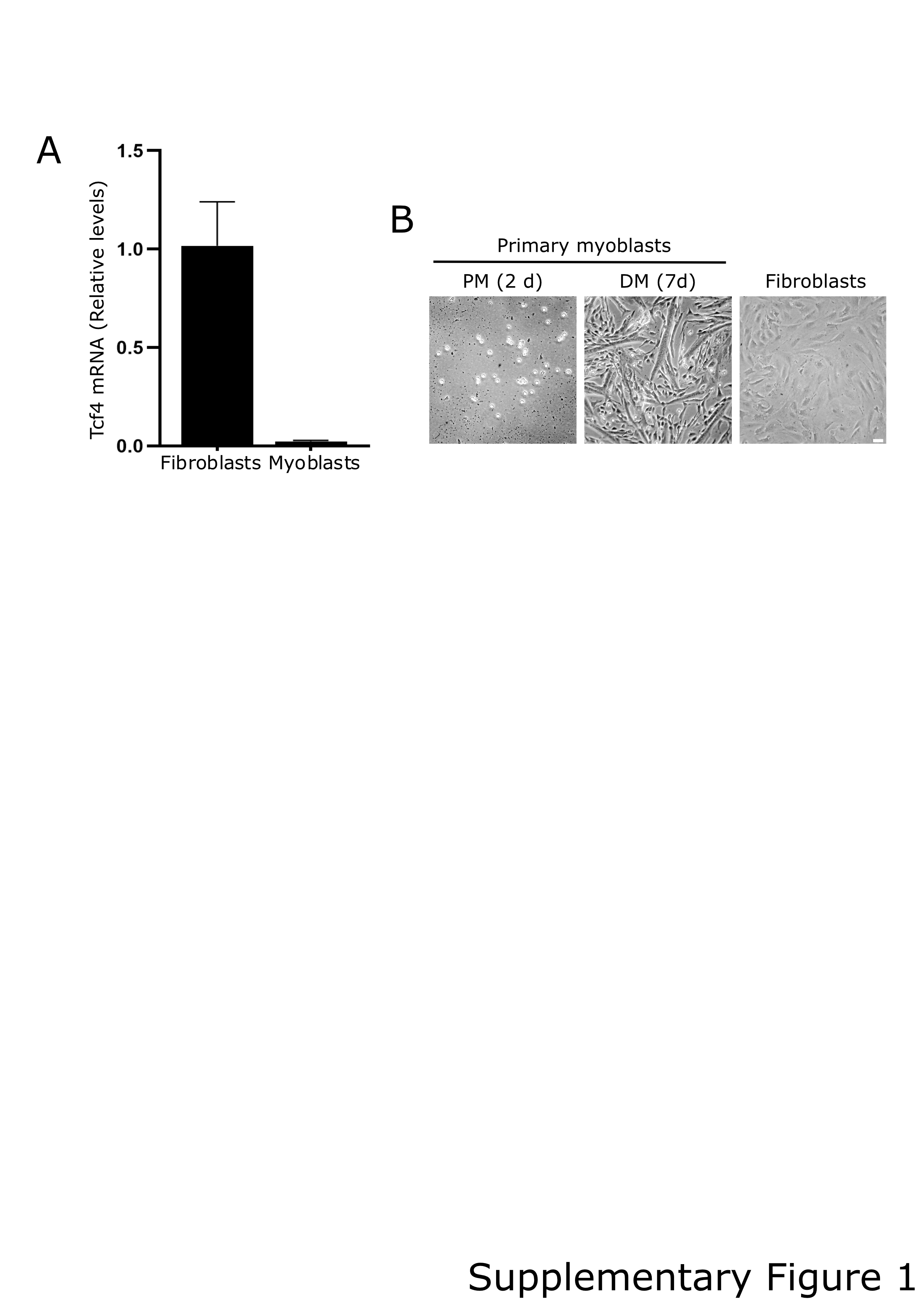

Supplement: Supplementary file 1 — Additional file 1: Figure S1. A) To determine the purity of SCs cultures, the fibroblast marker Tcf4 mRNA relative levels were measured by qPCR from SCs primary cultures maintained in differentiation conditions for 7 days and compared with fibroblasts cultures maintained for the same period. Results were expressed as the average RQ ± SD of three experimental replicates. B) Phase contrast images representative from primary cultures in. Scale bar = 20 μm. Representative of 3–4 independent experiments. [file 40659_2023_432_MOESM1_ESM.jpg]

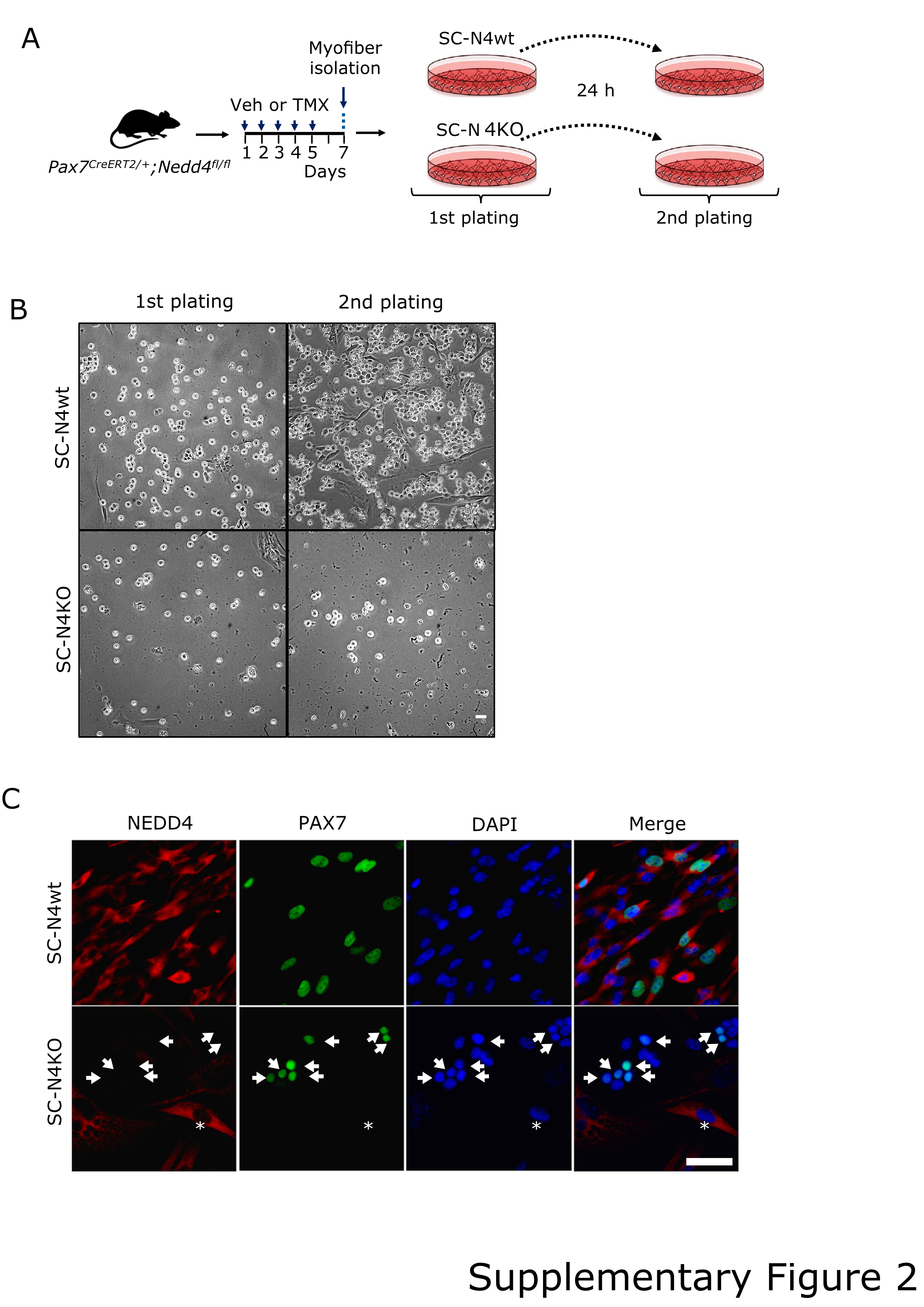

Supplement: Supplementary file 2 — Additional file 2: Figure S2. A) Pax7CreERT2/+; Nedd4-1f/f mice were injected with tamoxifenor vehiclefor 5 days. 48 h after the final dose, myofibers were isolated and maintained in proliferating culture conditions for 24 h. Subsequently, culture supernatant was transferred to a second plateand maintained in proliferating culture conditions for 24 h. B) Representative phase contrast microscopy from primary myoblasts obtained as in. n = 3. Scale bar = 10 μm. C) SCs were isolated from Pax7CreERT2/+; Nedd4-1f/f mice and treated with vehicleor TMX. After 72 h, cells were fixed, and NEDD4-1 expression was analyzed by IF. PAX7 expression was used as muscle progenitor marker. Nuclei were stained with DAPI. Arrows indicate NEDD4-1muscle precursors, and asterisk shows non-myogenic cell expressing NEDD4-1 in TMX-treated cultures. Scale bar = 50 μm. [file 40659_2023_432_MOESM2_ESM.jpg]

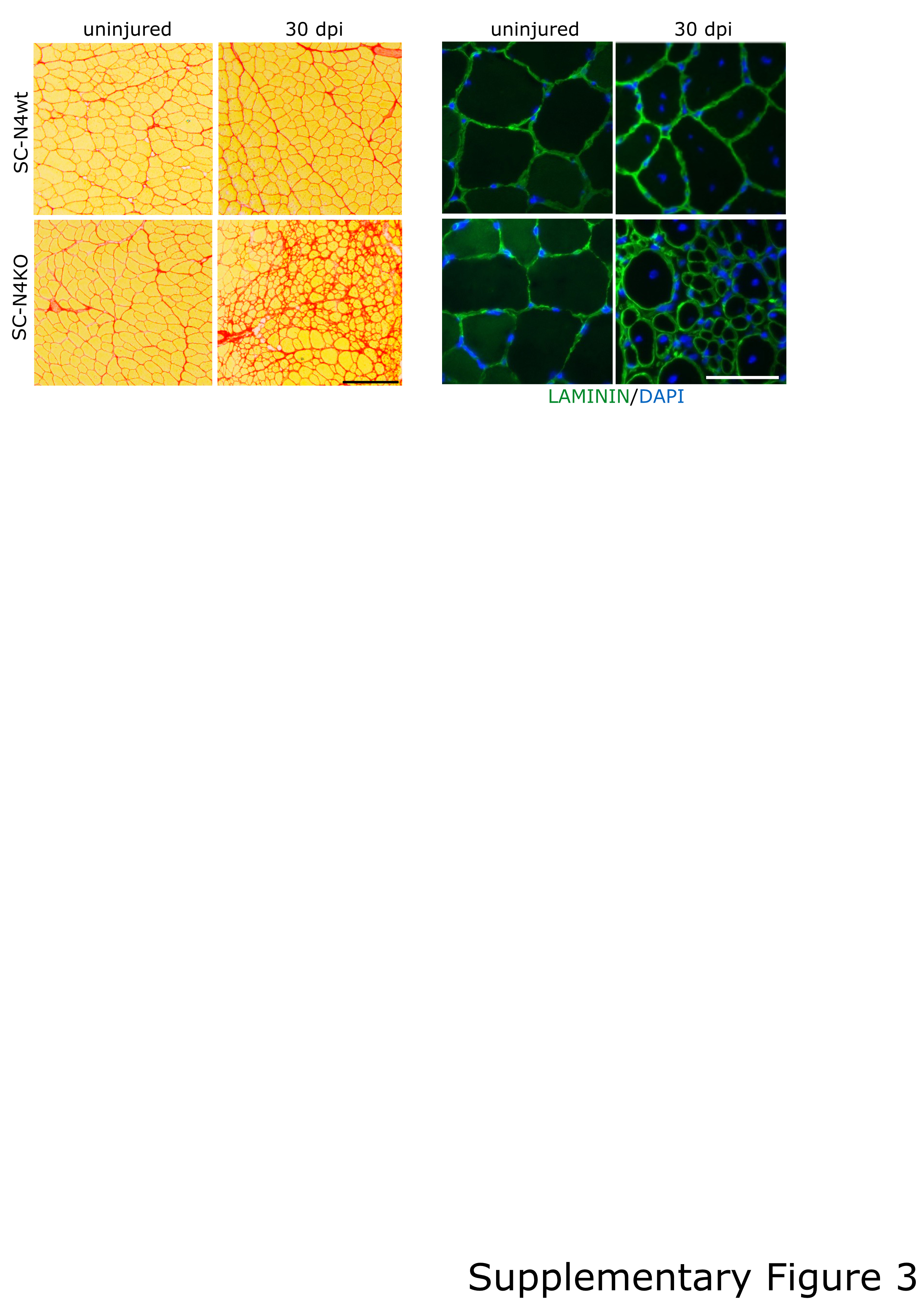

Supplement: Supplementary file 3 — Additional file 3: Figure S3. Sirius red staining and IF for lamininof contra-lateraland injuredTAs from SC-N4wt and SC-N4KO mice obtained as in Fig. 6. Scale bars = 200 μm, 50 μm. [file 40659_2023_432_MOESM3_ESM.jpg]

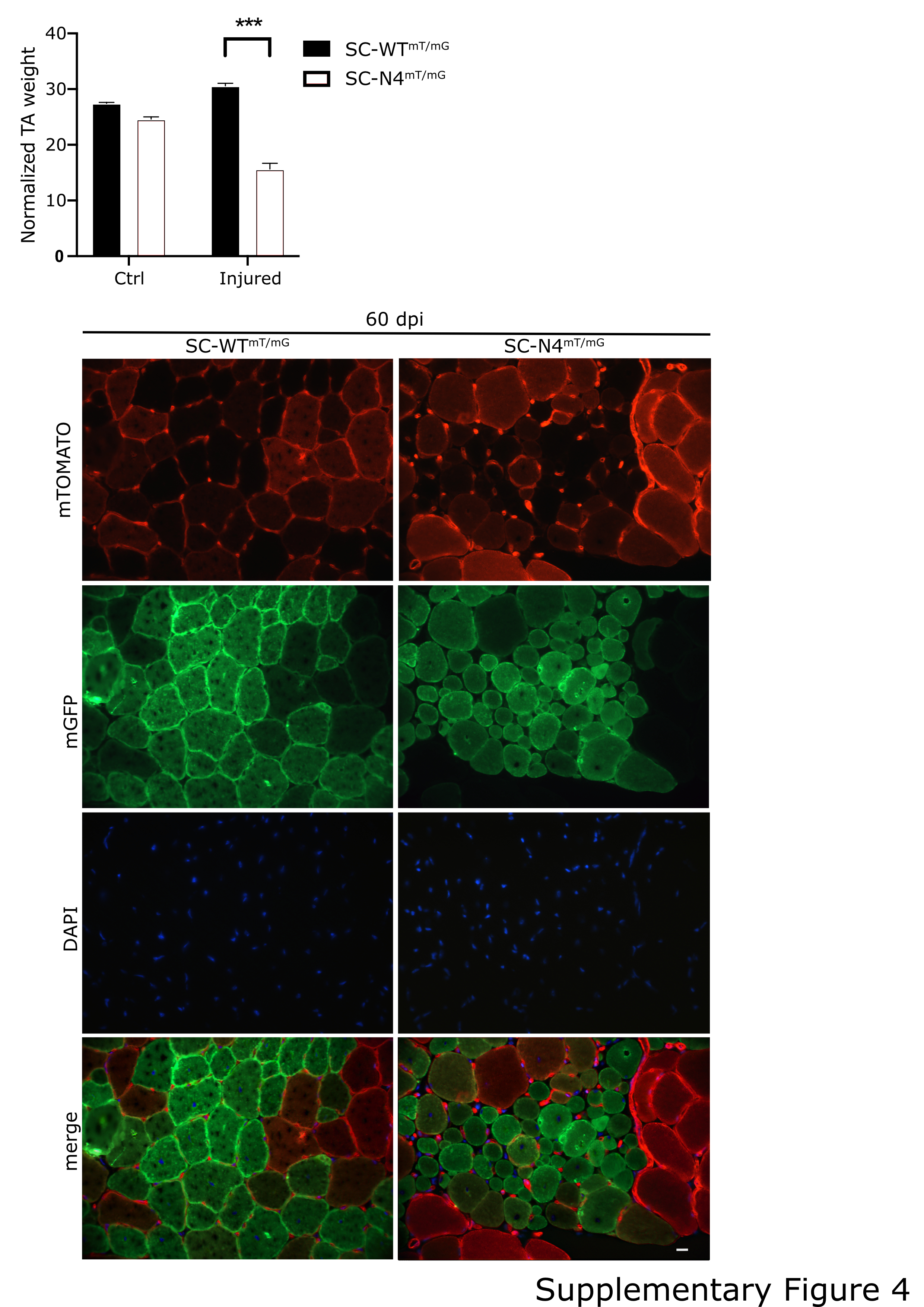

Supplement: Supplementary file 4 — Additional file 4: Figure S4. A) Normalized TA weightdifferences from SC-N4wtmT/mG and SC-N4KOmT/.mG at 60 dpi. *** P < 0.001. B) TA cryosections from SC-N4wtmT/mG and SC-N4KOmT/mG at 60 dpi. mGFPmyofibers show contribution of recombined SCs to muscle regeneration. Representative of n=3 for SC-WT and n=4 for SC-N4KO. Scale bar= 10 μm. [file 40659_2023_432_MOESM4_ESM.jpg]
